# The European Academy of Andrology (EAA) ultrasound study on healthy, fertile men: Prostate‐vesicular transrectal ultrasound reference ranges and associations with clinical, seminal and biochemical characteristics

**DOI:** 10.1111/andr.13217

**Published:** 2022-07-19

**Authors:** Francesco Lotti, Francesca Frizza, Giancarlo Balercia, Arcangelo Barbonetti, Hermann M. Behre, Aldo E. Calogero, Jann‐Frederik Cremers, Felice Francavilla, Andrea M. Isidori, Sabine Kliesch, Sandro La Vignera, Andrea Lenzi, Marios Marcou, Adrian Pilatz, Olev Poolamets, Margus Punab, Maria Fernanda Peraza Godoy, Claudia Quintian, Osvaldo Rajmil, Gianmaria Salvio, Osama Shaeer, Wolfgang Weidner, Elisa Maseroli, Sarah Cipriani, Elisabetta Baldi, Selene Degl'Innocenti, Giovanna Danza, Anna Lucia Caldini, Alessandro Terreni, Luca Boni, Csilla Krausz, Mario Maggi

**Affiliations:** ^1^ Andrology Female Endocrinology and Gender Incongruence Unit Department of Experimental and Clinical Biomedical Sciences “Mario Serio” University of Florence Florence Italy; ^2^ Endocrinology Unit Ospedali Riuniti Ancona Polytechnic University of Marche Ancona Italy; ^3^ Andrology Unit Department of Life Health and Environmental Sciences University of L'Aquila L'Aquila Italy; ^4^ Center for Reproductive Medicine and Andrology Martin Luther University Halle‐Wittenberg Halle Germany; ^5^ Department of Clinical and Experimental Medicine University of Catania Catania Italy; ^6^ Department of Clinical and Surgical Andrology Centre of Reproductive Medicine and Andrology Münster University Hospital Münster Germany; ^7^ Department of Experimental Medicine Sapienza University of Rome Rome Italy; ^8^ Department of Urology Pediatric Urology and Andrology Justus Liebig University Giessen Germany; ^9^ Andrology Unit Tartu University Hospital Tartu Estonia; ^10^ Andrology Department Fundació Puigvert, Universitat Autònoma de Barcelona Instituto de Investigaciones Biomédicas Sant Pau (IIB‐Sant Pau) Barcelona Spain; ^11^ Department of Andrology Kasr El Aini Faculty of Medicine Cairo University Cairo Egypt; ^12^ Endocrinology Unit Department of Experimental and Clinical Biomedical Sciences “Mario Serio” University of Florence Florence Italy; ^13^ Department of Laboratory Careggi Hospital Florence Italy; ^14^ Clinical Trials Coordinating Center Toscano Cancer Institute University Hospital Careggi Florence Italy

**Keywords:** clinical, seminal, hormonal and metabolic parameters, Healthy, fertile men, prostate and seminal vesicles reference ranges and normative parameters, transrectal ultrasound

## Abstract

**Background:**

Transrectal ultrasound (TRUS) parameters are not standardized, especially in men of reproductive age. Hence, the European Academy of Andrology (EAA) promoted a multicenter study to assess the TRUS characteristics of healthy‐fertile men (HFM) to establish normative parameters.

**Objectives:**

To report and discuss the prostate and seminal vesicles (SV) reference ranges and characteristics in HFM and their associations with clinical, seminal, biochemical parameters.

**Methods:**

188 men (35.6 ± 6.0 years) from a cohort of 248 HFM were studied, evaluating, on the same day, clinical, biochemical, seminal, TRUS parameters following Standard Operating Procedures.

**Results:**

TRUS reference ranges and characteristics of the prostate and SV of HFM are reported herein. The mean PV was ∼25 ml. PV lower and upper limits were 15 and 35 ml, defining prostate hypotrophy and enlargement, respectively. PV was positively associated with age, waistline, current smoking (but not with T levels), seminal volume (and negatively with seminal pH), prostate inhomogeneity, macrocalcifications, calcification size and prostate arterial parameters, SV volume before and after ejaculation, deferential and epididymal size. Prostate calcifications and inhomogeneity were frequent, while midline prostatic cysts were rare and small. Ejaculatory duct abnormalities were absent. Periprostatic venous plexus size was positively associated with prostate calcifications, SV volume and arterial peak systolic velocity. Lower and upper limits of SV anterior‐posterior diameter after ejaculation were 6 and 16 mm, defining SV hypotrophy or dilation, respectively. SV total volume before ejaculation and delta SV total volume (DSTV) positively correlated with ejaculate volume, and DSTV correlated positively with sperm progressive motility. SV total volume after ejaculation was associated negatively with SV ejection fraction and positively with distal ampullas size. SV US abnormalities were rare. No association between TRUS and time to pregnancy, number of children or history of miscarriage was observed.

**Conclusions:**

The present findings will help in better understanding male infertility pathophysiology and the meaning of specific TRUS findings.

## 1 INTRODUCTION

Transrectal ultrasound (TRUS) was developed in the second half of the fifties.^1^, ^2^, ^3^, ^4^ In 1955, Wild and Reid proposed a screw‐type transrectal radial scanner to investigate the intrapelvic organs.^2^, ^3^ However, TRUS was first applied to prostate examination in 1963 by Takahashi and Ouchi.^1^ Subsequently, Watanabe et al.^2^, ^3^ established the standards for imaging the prostate, seminal vesicles (SV) and other pelvic organs. In the mid‐eighties, TRUS was recognized as the best image modality of the prostate,^3^, ^4^ while more recently a systematic approach to SV imaging has been used.^5^ Nowadays, TRUS is considered superior to suprapubic ultrasound (US) to measure prostate volume^6^ and evaluate SV,^5^, ^7^ although it is minimally invasive. In addition, it is easier to perform, less expensive and less time consuming than other imaging techniques such as computed tomography and magnetic resonance.^7^

TRUS can detect alterations in size, echotexture and vascularization of the prostate and SV, therefore it is used to investigate several pathological conditions.^7^ Attempts to use TRUS to diagnose prostate cancer have been made over time, however, with scanty results, and currently it is not recommended for this purpose.^8^ On the other hand, TRUS has become increasingly relevant in investigating male reproductive and general health disturbances.^6^, ^7^, ^9^, ^10^ In fact, TRUS can be used to assess obstructive azoospermia^7^, ^9^ and SV abnormalities/agenesis.^7^, ^9^ In addition, it is useful in measuring prostate volume in relation to lower urinary tract symptoms, predicting their progression and risk of complications.^6^ Furthermore, recent evidence supports the use of TRUS to evaluate prostate inflammation,^7^, ^11^, ^12^, ^13^ related acquired premature ejaculation,^7^, ^13^, ^14^ chronic pelvic^7^, ^11^, ^12^, ^13^, ^15^ and post‐ejaculatory^15^ pain. Moreover, TRUS offers indirect information on male androgenization by assessing the size of the prostate and SV, androgen‐dependent glands which are reduced in hypogonadal men.^16^, ^17^, ^18^ Finally, it can be used to evaluate the prostate‐vesicular response to hormonal treatments.^16^, ^17^, ^18^

Although TRUS is widely used to explore the prostate‐vesicular region, there is still no consensus on the method used to assess several qualitative and quantitative colour‐Doppler US (CDUS) parameters.^7^ Furthermore, TRUS normative parameters and the cut‐offs for distinguishing between normal and pathologic features are still lacking.^7^ Finally, the possible correlation/impact of several TRUS findings on semen parameters and male fertility is still unclear.^7^ Due to the lack of male genital tract (MGT)‐CDUS standardization, the European Academy of Andrology (EAA) has promoted an international multicenter study entitled “Standardization of the MGT‐CDUS parameters in healthy, fertile men” (shortened to “EAA US study”; see http://www.andrologyacademy.net/studies)^19^ aimed at establishing a cohort of healthy, fertile men as a reference point for defining MGT‐CDUS normative parameters. In a previous study^20^ of a cohort of 248 healthy, fertile men, we described: (i) the development and methodology of the EAA US study, (ii) the clinical, seminal and biochemical parameters of the cohort and (iii) the correlations of both fertility history and seminal features with the aforementioned parameters. In particular, we reported that the seminal characteristics of the population studied were consistent with those reported by the WHO^21^ for the 50th and 5th centile for fertile men, identifying the EAA cohort as a reference point for assessing MGT‐CDUS normative parameters.^20^ In a subsequent study,^22^ we reported the reference ranges and characteristics of the scrotal organs in healthy, fertile men and their associations with clinical, seminal and biochemical parameters.

In the present study, we report and discuss the prostate and SV reference ranges and characteristics assessed by TRUS in healthy, fertile men, and their associations with clinical, seminal and biochemical parameters.

## 2 METHODS

The EAA US study was designed as a multicenter, international, observational study.^20^ Eleven EAA Centers (Ancona, Italy; Barcelona, Spain; Cairo, Egypt; Catania, Italy; Florence, Italy; Giessen, Germany; Halle, Germany; L'Aquila, Italy; Muenster, Germany; Rome, Italy; Tartu, Estonia) joined the project and enrolled 248 healthy, fertile men from February 2016 to February 2019. The definition of “healthy, fertile men” established by the EAA consortium has been reported and discussed in a previous study.^20^ The inclusion criteria of the EAA US study^20^ were: 1. healthy, fertile men (see below); 2. age ≥ 18 years; 3. capacity to give consent for study participation. “Fertile men” were defined as (i) partners of a pregnant woman in the second or third trimester of pregnancy or (ii) men with a child less than one year old, achieved through natural conception.^20^ “Healthy men” were defined as subjects with no personal history of previous or current systemic diseases or treatments with a recognized negative effect on semen parameters.^20^ All subjects were asked to undergo a standardized protocol performed entirely in the same day, including: scrotal and transrectal CDUS before and after ejaculation; personal and medical history and physical examination; blood sampling for the determination of biochemical parameters; semen analysis.^20^ Of the 248 subjects enrolled,^20^ 188 men (see below, “Results section”) accepted to undergo TRUS before and after ejaculation. The Standard Operating Procedures (SOPs) for the assessment of TRUS qualitative and quantitative parameters and the intra‐ and inter‐operator comparability of the MGT‐CDUS parameters among different operators have been defined during investigator meetings organized before starting the enrollment of healthy, fertile men, as previously reported,^20^ and are extensively described below. The SOPs for the assessment of scrotal CDUS have been extensively described in a previous study.^22^

### 2.1 Clinical, biochemical and seminal parameters

The methods related to the clinical, seminal and biochemical parameters of the cohort studied have been reported and discussed in a previous study.^20^ In particular, general and andrological physical examinations were carefully performed according to previous reports.^20^

### 2.2 SOPs to assess TRUS qualitative and quantitative parameters

**FIGURE 1:**
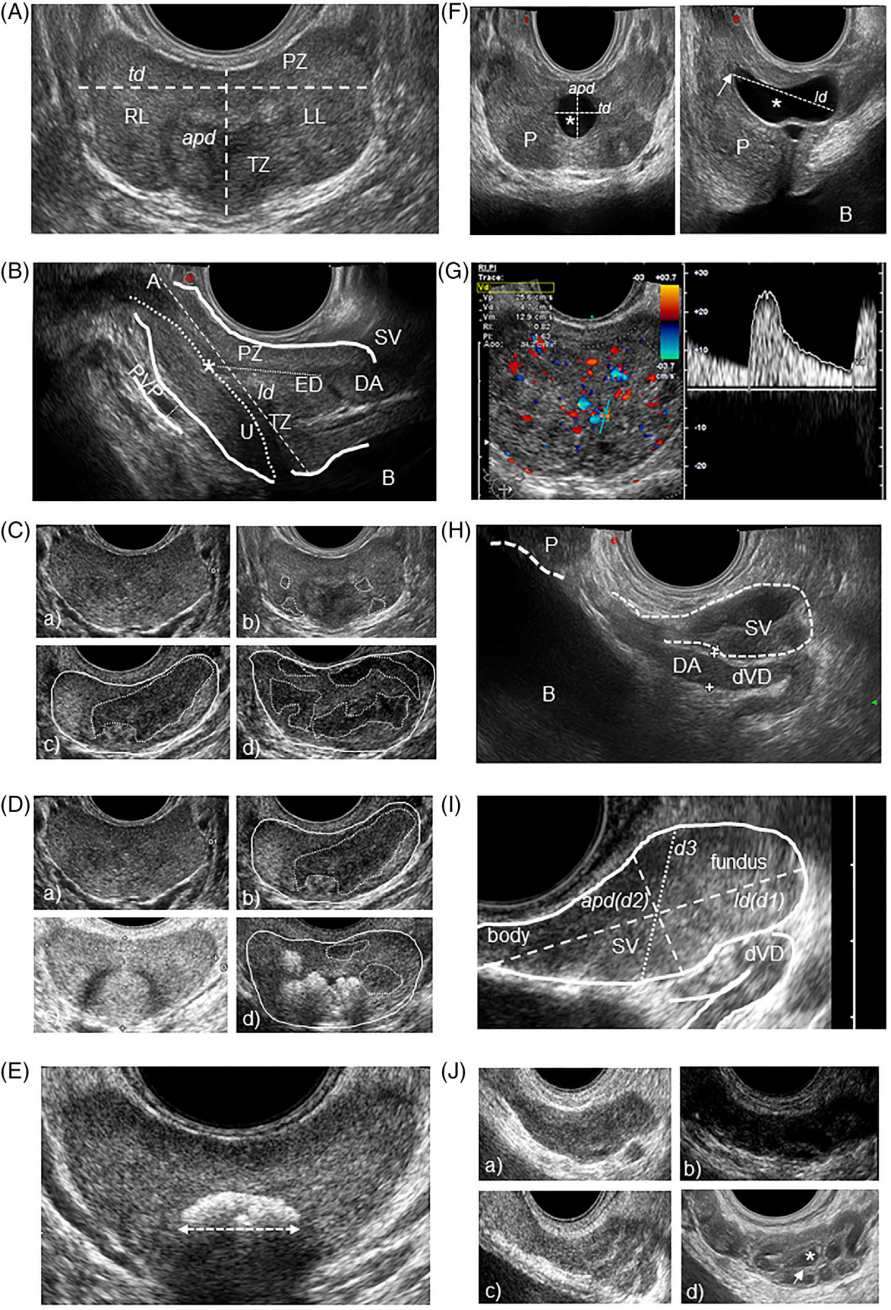
Standard Operating Procedures (SOPs) for the assessment of the main transrectal ultrasound (TRUS) prostate (P) and seminal vesicles (SV) qualitative and quantitative parameters. The TRUS parameters analyzed and the methods used to evaluate them are extensively reported and discussed in the main text and at http://www.andrologyacademy.net/studies19. **(A**) Prostate of normal volume, homogeneity and echogenicity in transversal scan. Peripheral and transitional zone (PZ and TZ) show a 3:1 ratio in young men.^7^ Right and left lobes (RL and LL, respectively) are indicated. Anterior‐posterior and transverse diameters (*apd* and *td*, respectively) are reported as dashed lines. **(B**) Prostate of normal volume, homogeneity and echogenicity in sagittal scan evaluated with “end fire” probe. Peripheral and transitional zone (PZ and TZ, respectively) and apex (A) are indicated, as well as bladder (B), urethra (U, dotted line), ejaculatory duct (ED, small dots line), prostatic utricle (*), deferential ampulla (DA), SV and a section of periprostatic venous plexus (PVP), the size of which is defined with small dots. The longitudinal diameter (*ld*) is reported and represented as a dashed line. **(C**) Prostate homogeneity/inhomogeneity degrees, classified on a four point‐Likert scale: a) homogeneity; b) mild (grade 1) inhomogeneity [presence of small hypo‐ or hyper‐echoic foci]; c) moderate (grade 2) inhomogeneity [presence of large hypo‐ or hyper‐echoic areas]; d) severe (grade 3) inhomogeneity [diffuse inhomogeneity with “geographical map” appearance]). Hypoechoic areas are demarcated with dotted lines. **(D**) Prostate echogenicity, classified on a four point‐scale: a) 0.normal echogenicity; b) 1.mainly hypoechoic/presence of large hypoechoic areas; c) 2.mainly hyperechoic/presence of large hyperechoic areas; d) 3.mixed pattern [diffuse hypo‐ and hyper‐echoic areas], with hypoechoic areas demarcated with dotted lines and markedly hyper‐echoic areas represented by calcifications. (**E)** Prostate in transversal scan with a central macrocalcification, the major diameter of which is measured. **(F)** Midline prostatic cyst (*) in transversal (left) and sagittal (right) scan. The cyst diameters (anterior‐posterior [*apd*], transverse [*td*] and longitudinal [*ld*] diameters) are reported as dashed lines. The prostatic utricle is indicated with an arrow. P, prostate; B, bladder. **(G)** Prostate arterial vascularization, with hyperemia and measurement, in the transitional zone, of arterial parameters including peak systolic velocity (Vp), resistive index (RI), pulsatility index (PI) and acceleration (Acc.). **(H)** Distal vas deferens (dVD) and deferential ampulla (DA) beside a section of the seminal vesicle (SV) assessed by “end fire” probe in sagittal scan. Bladder (B) and prostate (P) are visible. DA size is demarcated by two crosses. **(I)** Seminal vesicle (SV) assessed by “end fire” probe in sagittal scan. SV fundus and body are reported, as well as maximum longitudinal and anterior‐posterior diameters (*ld* and *apd* dashed lines, respectively). SV volume has been calculated (see the main text) using the ‘ellipsoid/prolate spheroid (d1 > d2 = d3)’ mathematical formula considering d1 = *ld*, d2 = *apd*, and d3 = d2 (dotted line).^5^ dVD, distal vas deferens. **(J**) SV echotexture features: (i) homogeneity (a) / inhomogeneity (d) and (ii) echogenicity, classified on a four point‐scale: (a) 0.normal echogenicity; (b) 1.mainly hypoechoic/presence of large hypoechoic areas; (c) 2.mainly hyperechoic/presence of large hyperechoic areas; d) 3.mixed pattern (diffuse hypo‐ and hyper‐echoic areas), represented by a SV with roundish anechoic areas (*) and thickened septa (arrow)

The TRUS parameters to be analyzed and the methods used to evaluate them were standardized and reported at http://www.andrologyacademy.net/studies19. In addition, exemplary figures reporting (a) how to measure quantitative parameters and (b) classifications of qualitative characteristics – using Likert scales ‐ of the prostate and SV were reported on the EAA website,^19^ and Figure 1 shows the most relevant figures. Finally, standardized schedules to report parameters detected before and after ejaculation in each EAA Center were uploaded and made available at http://www.andrologyacademy.net/studies19.

### 2.3 TRUS

TRUS has been performed systematically on the subjects studied scanning the organs at 5 mm intervals at various longitudinal, transverse and oblique scans with the patients placed in the left lateral decubitus using a transrectal probe (3‐13 MHz).^7^ The ultrasonographic equipments used by the different EAA Centers are showed in a Supplementary Table.

### 2.4 Prostate

Prostate volume (PV) was assessed using the planimetric method,^7^, ^16^, ^23^ by measuring the maximum anterior‐posterior and transverse diameters (*apd* and *td*, respectively) in a transversal scan (Figure 1A) and the maximum longitudinal diameter (*ld*) in a longitudinal scan (Figure 1B), and calculating the volume using the ellipsoid formula (*apd* x *td* x *ld* x π/6).^23^, ^24^, ^25^, ^26^ Similar methods were also used to determine the transitional zone volume and adenoma volume, when present.^24^, ^25^, ^26^ The EAA consortium stated that the *apd* and the *td* had to be measured in two different subsequent transrectal scans, allowing to measure the maximum *apd* and the maximum *td*, respectively. The maximum *td* has been defined as the maximum distance between the lateral margins of the prostatic lobes, measured in a freezed scan showing the largest transversal prostate section/area (Figure 1A). The maximum *apd* has been defined as the maximum midline distance between the posterior margin of the prostate (peripheral zone) and the anterior margin of the transitional zone (including the smooth sphincter of the urethra) (Figure 1A). The maximum *ld* had to be measured in a longitudinal scan, on the midline sagittal plane, from the base of the prostate, in a position immediately posterior to the origin of the prostatic urethra / bladder neck, to the apex of the prostate, viewable immediately paramedian to the striated sphincter of the urethra (Figure 1B).

Prostate symmetry^27^, ^28^ was classified as a dummy variable (0. symmetric [Figure 1A]; 1. asymmetric). Asymmetry was defined when the difference (mm) between the right and left prostate lobes (evaluated in a transversal scan from midline to lateral margin) was greater than the 95th percentile of its distribution (see below, “Statistical analysis”). In the event of asymmetry, the side of the bigger lobe and the length difference (mm) between lobes was reported.

Prostate homogeneity was classified by the EAA US consortium on a four point‐Likert scale (0. homogeneity; 1: mild (grade 1) inhomogeneity [presence of small hypo‐ or hyper‐echoic foci]; 2: moderate (grade 2) inhomogeneity [presence of large hypo‐ or hyper‐echoic areas]; 3: severe (grade 3) inhomogeneity [diffuse inhomogeneity with “geographical map” appearance]), according to a previous study^29^ (Figure 1C).

Prostate echogenicity was classified by the EAA US consortium on a four point‐scale (0: normal echogenicity; 1: mainly hypoechoic/presence of large hypoechoic areas; 2: mainly hyperechoic/presence of large hyperechoic areas; 3: mixed [diffuse hypo‐ and hyper‐echoic areas]), resembling previous studies^23^, ^27^, ^28^ (Figure 1D).

Size and location of calcifications were assessed, and the maximum diameter of the major calcifications (during transversal or longitudinal scan) has been reported^23^ (Figure 1E). Macrocalcifications were defined as > 3 mm, according to a previous report^23^(Figure 1E). Microcalcifications were defined as small (1–3 mm) echogenic foci. The location of the calcifications was reported considering (i) three arbitrary virtual areas: upper, middle and lower third of the prostate in a longitudinal scan, and (ii) if they were in the transitional or peripheral zone, unilateral or bilateral, isolated or multiple, in a trasversal scan (Figure 1E).

Midline prostatic cysts were defined as midline roundish or pear/oval‐shaped anechoic formations, and, when present, their diameters were measured (Figure 1F) and volume calculated using the ellipsoid formula.^30^ In addition, non‐midline prostatic cysts were investigated.^30^ The presence of prostate nodules, especially in the peripheral zone, was also investigated.^8^

Prostate‐related vascular parameters were evaluated before and after ejaculation. Arterial parameters were assessed in the transitional/urethral and capsular zones,^31^, ^32^ including arterial peak systolic velocity (PSV),^12^, ^13^, ^33^ acceleration,^34^ resistive index (RI)^32^, ^33^, ^35^ and pulsatility index (PI)^36^ (Figure 1G), and reported as the mean value of three different measurements. The angle of insonation used was between 40 and 60° and angle correction followed the vascular flow direction.^37^ The EAA consortium defined hyperaemia as a diffuse increase of prostate arterial vascularization^11^, ^12^, ^13^ (Figure 1G), suggesting to detect ≥ 15 colour‐Doppler spots according to a previous study^11^ in an attempt to move from a qualitative to a quantitative assessment, although aware that different US equipements may have different sensitivity in colour‐Doppler spots revealing. Periprostatic venous plexus (PVP) was evaluated^14^, ^23^ measuring the maximum *apd* in a longitudinal scan (Figure 1B) and its flux velocity.

### 2.5 Ejaculatory ducts and deferential ampullas

The characteristics of ejaculatory ducts (Figure 1B) and deferential ampullas (Figure 1B and H) were evaluated before and after ejaculation, the latter to emphasize more clearly any possible indirect US signs of partial or complete obstruction.^7^ Ejaculatory duct abnormalities, including dilation (> 2 mm), calcifications or cysts,^7^ were investigated, and classified as 0: absent, 1: unilateral or 2: bilateral. Deferential ampullas were investigated and scored 0: presence, 1: unilateral absence or 2: bilateral absence; their maximum *apd* was measured during a longitudinal scan near insertion into the prostate^7^, ^38^ (Figure 1H).

### 2.6 Seminal vesicles (SV)

SV were evaluated before and after ejaculation,^5^, ^7^ and were scored 0: presence, 1: unilateral absence or 2: bilateral absence. For each SV, the maximum *ld* and *apd* were measured before and after ejaculation^5^, ^7^, ^39^, ^40^, ^41^ in a longitudinal scan (Figure 1I). The EAA consortium stated that the maximum SV diameters had to be measured in a freezed sagittal scan showing the largest SV area, defining *ld* as the distance from the superior pole of the SV fundus to the insertion of the SV body into the prostate (Figure 1I) and *apd* as the maximum distance between the lateral margins of the SV fundus (Figure 1I). SV volume was calculated using the “ellipsoid/prolate (d1 > d2 = d3) spheroid” mathematical formula (d1 × d2 × d3 × 4/3 × π, considering d1 = 1/2 the maximum SV‐*ld*, d2 = 1/2 the maximum *apd* and d3 = d2)^5^, ^7^, ^39^, ^40^, ^41^ (Figure 1I). “SV total volume” was calculated as the sum of right and left SV volume. “Delta SV total volume” (DSTV) was calculated as SV total volume before ejaculation ‐ SV total volume after ejaculation,^42^ and delta SV diameters was calculated accordingly. “SV ejection fraction” was calculated as: [(DSTV) / SV total volume before ejaculation] × 100.^5^, ^7^, ^39^

SV symmetry^27^, ^28^ was classified as a dummy variable (0: symmetric, 1: asymmetric). Asymmetry was defined when the difference between right and left SV‐*apd*, *ld* (mm) or volume (ml) was greater than the 95th percentile of its distribution (see below, “statistical analysis”).

SV homogeneity^5^, ^7^ was classified as a dummy variable (0: homogeneous; 1: inhomogeneous) (Figure 1J). SV echogenicity^7^, ^23^, ^40^ was classified by the EAA US consortium on a four point‐scale (0: normal echogenicity; 1: mainly hypoechoic/presence of large hypoechoic areas; 2: mainly hyperechoic/presence of large hyperechoic areas; 3: mixed [diffuse hypo‐ and hyper‐echoic areas]) (Figure 1J).

SV‐US abnormalities were investigated and classified as 0: absent, 1: unilateral or 2: bilateral, including areas of endocapsulation/roundish anechoic areas^5^, ^7^, ^27^, ^28^, ^43^ (Figure 1J), wall thickening and septa^5^, ^7^, ^27^, ^28^, ^43^ (Figure 1J), calcifications^7^ and giant cysts.^5^, ^7^, ^43^ SV arterial parameters, including PSV, acceleration, RI and PI, were measured before and after ejaculation assessing CDUS spots detected in the SV wall.^31^, ^45^

### 2.7 Intra‐ and inter‐operator comparability of TRUS parameters

During the third EAA investigator meeting, held in Florence on April 20, 2013,^20^ intra‐ and inter‐operator comparability of the MGT‐CDUS parameters were assessed on seven males of infertile couples.^20^, ^22^ Intra‐operator comparability was assessed for the main quantitative and qualitative TRUS parameters considering the results of three evaluations for each parameter.^22^ Inter‐operator comparability was derived from the measures and observations obtained by six different sonographists (F.L., F.F., O.P., G.S., E.M., S.C.) for the main quantitative and qualitative parameters, respectively.^22^ The comparability of quantitative and qualitative parameters was expressed using the coefficient of variation (CV) [(standard deviation (*σ*) / mean (*μ*)) x 100] and the concordance rate (CR) [(number of concordant observations/number of operators) x 100)], respectively.^22^, ^46^ A CV < 10 is considered acceptable.^22^, ^47^

### 2.8 Satistical analysis

Data are expressed as mean ± SD when normally distributed, as medians (quartiles) for parameters with non‐normal distribution, and as percentages when categorical. The reference range for prostate‐vesicular organs was estimated according to the Clinical and Laboratory Standard Institute (CLSI) Guidelines,^22^, ^48^ as the 5th and the 95th percentiles of its distribution. Correlations were assessed using Spearman's or Pearson's method, whenever appropriate. Stepwise multiple linear or logistic binary regressions were applied for multivariate analyses, whenever appropriate. When distribution could be normalized through logarithmic transformation, the same test was applied to logarithmically transformed data. For continuous parameters, a comparison between two groups in a univariate setting was performed, with unpaired two‐sided Student's t tests for variables with normal distribution or Mann–Whitney U‐test for variables with not normal distribution, and analysis of covariance (ANCOVA) was used for comparisons between two groups in a multivariate setting. Relative risk and 95% confidence interval were calculated for association of categorical parameters, and chi‐squared test was used for comparisons, using the Fisher's exact test whenever appropriate. Multivariate analyses of categorical parameters were performed using a binary logistic regression model. Multivariate analyses were performed adjusting for confounders including male age, waistline, smoking habit, alcohol consumption, physical activity, calculated free testosterone (T) levels and number (#) of EAA Centers (“adjusted model”), unless otherwise specified, according to previous studies.^20^, ^22^ In particular, current smoking, alcohol consumption and physical activity were codified as dummy variables 0–1 (no/yes), according to previous studies.^20^, ^22^ The paired two‐sided Student's t‐test was used to compare TRUS parameters evaluated before and after ejaculation. All statistical analysis was performed on SPSS (Statistical Package for the Social Sciences, Chicago, IL, USA) for Windows 26.0. A p<0.05 was considered as significant.

## 3 RESULTS

Out of 248 healthy, fertile subjects enrolled in the EAA US study,^20^ 188 (35.6 ± 6.0 years; range 23–53 years) underwent TRUS before and after ejaculation from February 2016 to February 2019. The socio‐demographic, clinical, seminal, biochemical and scrotal CDUS characteristics of the entire cohort (*n* = 248) have been reported in previous studies.^20^, ^22^ Table 1 shows the main clinical characteristics of the subjects (*n* = 188) who underwent TRUS, which are comparable to those of the entire sample.^20^ Complete US data on prostate and SV were available for 188 and 142 men, respectively.

**TABLE 1:** Clinical parameters of the sample. Data were expressed as mean ± SD when normally distributed, as medians (quartiles) for parameters with non‐normal distribution, and as percentages when categorical. BP, blood pressure; T, testosterone; HPLC‐MS, high performance liquid chromatography tandem mass spectrometry

Socio‐demographic parameters	*n* = 188
Age (years)	35.6 ± 6.0
Physical activity (%)	53.2
Current smokers (%)	21.8
Current alcohol consumption (%)	34.5
**History of genito‐urinary infections (%)**	18.1
Prostatitis	6.9
**Seminal parameters**	
Sexual abstinence (days)	4.0 ± 1.3
pH	7.7 ± 0.2
Semen volume (ml)	3.1 ± 1.7
Sperm concentration, *10^6^/ml	71.0 (45.5–120.0)
Sperm total count, *10^6^/ejaculate	207.0 (112.0–333.0)
Sperm progressive motility, %	53.0 ± 14.0
Sperm morphology, % normal forms	8.0 (6.0–12.0)
Sperm vitality (%)	76.0 ± 11.0
Leukocytospermia (%)	7.5
Bacteriospermia (%)	6.4
Abnormal viscosity (%)	27.3
**Physical examination**	
Systolic BP (mm Hg)	122.0 ± 11.0
Diastolic BP (mm Hg)	79.0 ± 7.0
Body mass index (BMI, kg/m^2^)	24.8 ± 3.2
Waistline (cm)	92.5 ± 9.3
Mean testis volume (Prader) (ml)	21.1 ± 4.0
Varicocele (%)	25.0
Enlarged prostate at digito‐rectal examination (%)	5.9
**Biochemical parameters**	
FSH (U/l)	3.5 (2.3–4.8)
LH (U/l)	3.1 (2.4–4.5)
Total testosterone (nmol/l) (HPLC‐MS method)^20^	19.6 ± 7.5
Sex hormone binding globulin (nmol/l)	37.6 ± 15.2
Calculated free testosterone (cFT; pmol/l)^20^	395.0 ± 144.0
PSA (ng/ml)	0.77 (0.54–1.07)

### 3.1 Intra‐ and inter‐operator comparability of TRUS parameters

Table 2 shows the intra‐ and inter‐operator comparability of the main TRUS parameters, reporting the coefficient of variation for quantitative parameters and the concordance rate between operators for qualitative parameters.

**TABLE 2:** Intra‐ and inter‐operator comparability of the main TRUS parameters. Data are derived from the evaluation of seven males of infertile couples. Inter‐operator comparability has been obtained from the measures and observations of six different sonographers. CV = coefficient of variation (standard deviation [*σ*] / mean [*μ*] x 100). A CV < 10 is considered acceptable^47^. CR = concordance rate, ([number of concordant observations/number of operators] × 100)

	Intra‐operator comparability	Inter‐operator comparability
Prostate volume (ml)	CV = 0.84	CV = 7.65
Prostate inhomogeneity (yes/no)	CR = 100%	CR = 83.3%
Prostate calcifications (yes/no)	CR = 100%	CR = 100%
Prostate artery peak systolic velocity (cm/s)	CV = 3.08	CV = 8.03
Periprostatic venous plexus (mm)	CV = 3.22	CV = 7.75
Seminal vesicles total volume before ejaculation (ml)	CV = 6.80	CV = 9.67
Seminal vesicles total volume after ejaculation (ml)	CV = 6.72	CV = 9.56
Areas of endocapsulation before ejaculation (yes/no)	CR = 100%	CR = 83.3%
Areas of endocapsulation after ejaculation (yes/no)	CR = 100%	CR = 83.3%
Vas deferens ampulla size (mm)	CV = 3.47	CV = 7.87

### 3.2 Reference ranges of TRUS parameters

**FIGURE 2:**
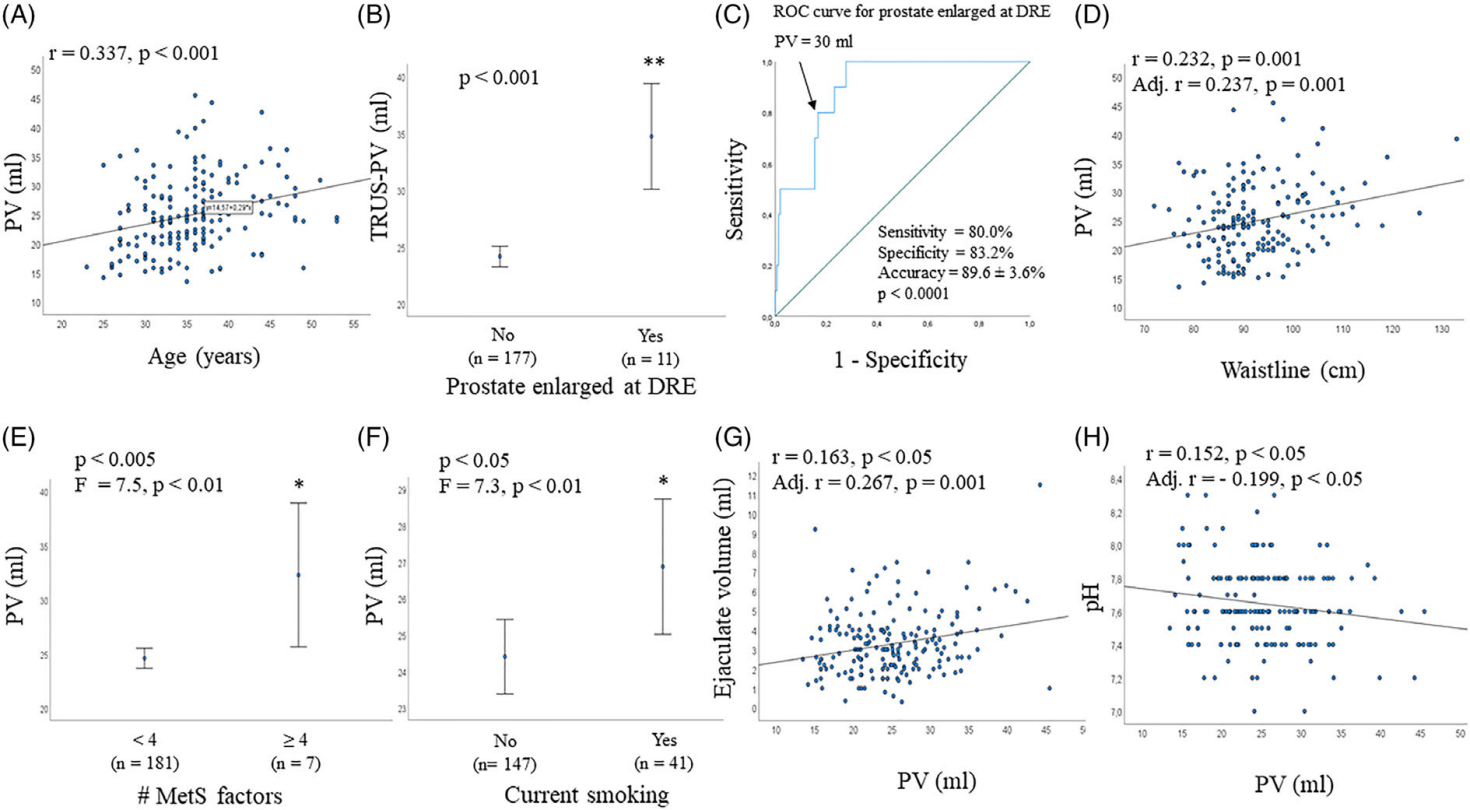
Associations between prostate volume (PV) at transrectal ultrasound (TRUS), clinical and seminal parameters. (A) association between PV and age. The equation of the relationship between age and PV (*y* = 14.57 + 0.29*x) is reported. (B) Association between PV at digito‐rectal examination (DRE) and TRUS‐PV. C, receiver operating characteristic (ROC) curve for PV as a function of increased prostate at DRE. (D–F) Associations between PV and waistline (C), number (#) of metabolic syndrome (MetS [AHA/NHLBI classification]^20^) factors (E), current smoking (F). (G–H) Associations between PV and seminal volume (G) and pH (H). (D–H) unadjusted and adjusted (for age, waistline, smoking habit, alcohol consumption, physical activity, cFT levels and # EAA Centers, when appropriate) associations have been reported. *p < 0.005; **p < 0.001 (unadjusted)

Table 3 shows the reference ranges of the prostate‐related quantitative parameters (including prostate diameters and volume, arterial PSV, acceleration, PI and RI and periprostatic venous plexus [PVP] size and flux velocity) and prostate echotexture characteristics, evaluated before and after ejaculation. The mean PV was 25.0 ± 6.3 ml, with a lower and higher limit of 15.0 and 35.0 ml, respectively. Similar figures were observed when a selected series (*n* = 141) of eugonadal men (cFT ≥ 225 pM)^20^ without central obesity (waistline ≤ 102 cm)^20^ was considered. Considering the relationship between age and PV (*y* = 14.57 + 0.29*x) (Figure 2A), age‐adjusted PV can be easily derived. By simplifying the equation, the age‐adjusted PV in healthy, fertile men under the age of 53 is equal to “1/3 age + 15”. Accordingly, the average PV categorized by age decades is reported in Table 3.

**TABLE 3:** Reference range and mean or median values and percentages of the prostate‐related color‐Doppler ultrasound (CDUS) parameters in healthy, fertile men. Data are expressed as mean ± SD when normally distributed, as medians (quartiles) for parameters with non‐normal distribution, and as percentages when categorical. The reference range of each parameter has been estimated according to the CLSI Guidelines^48^ as the 5th and the 95th percentiles of its distribution. US, ultrasound; CDUS, colour‐Doppler ultrasound; PSV, peak systolic velocity; RI, resistive index; PI, pulsatility index. *Eugonadal (cFT ≥ 225 pM)^20^ without central obesity (waistline < = 102 cm)^20^. **Macrocalcifications^23^ were isolated or multiple in 33% and 67% of cases, respectively; unilateral or bilateral in 70% and 30% of cases, respectively. The major calcification was located in the upper, middle and lower third of the prostate in 31%, 41% and 28% of cases, respectively, and located in the transitional or peripheral zone in 65% and 35% of cases, respectively

Prostate US parameters	Mean or median values and percentages	Reference range
Diameters (mm)		
Transversal (*td*)	45.0 ± 4.4	38.0–52.5
Anterior‐posterior (*apd*)	25.5 ± 3.7	18.0–31.0
Longitudinal (*ld*)	42.0 ± 4.3	34.0–49.0
Volume (ml)	25.0 ± 6.3	15.0–35.0
third age decade (23–30 years) (*n* = 36)	22.0 ± 6.0	15.0–34.0
fourth age decade (31–40 years) (*n* = 115)	25.0 ± 6.3	16.0–35.0
fifth age decade (41–53 years) (*n* = 37)	27.0 ± 5.6	18.0–37.0
Volume (ml) in eugonadal men without central obesity* (n=141)	24.5 ± 6.4	15.0–35.0
Asymmetry (%)	0.0	
Homogeneity (%)		
homogeneous (grade 0)	65.4	
mild inhomogeneity (grade 1)	29.8	
moderate inhomogeneity (grade 2)	4.8	
severe inhomogeneity (grade 3)	0.0	
Echogenicity (%)		
normoechoic	87.8	
mainly hypoechoic	6.4	
mainly hyperechoic	0.5	
mixed	5.3	
Calcifications (%)	42.5	
Micro‐calcifications (1‐3 mm) (%)	9.0	
Macro‐calcifications (> 3 mm)** (%)	33.5	
Major calcification diameter (mm)	7.5 (4.2–12.0)	3.0–18.0
Midline prostatic cyst (%)	5.0	
Transversal diameter (mm)	4.0 (3.25–4.75)	3.0–5.0
Anterior‐posterior diameter (mm)	3.0 (2.25–4.75)	2.0–6.0
Longitudinal diameter (mm)	6.0 (4.0–7.5)	4.0–9.0
Volume (ml)	0.038 (0.026–0.069)	0.012–0.117
Parenchymal cysts (%)	3.2	
maximum diameter (mm)	4.5 ± 2.1	2.0–7.0
Ejaculatory ducts		
dilation (> 2 mm)	0.5	
cysts	0.0	
micro‐calcifications	0.0	
Peripheral nodules	0.0	
**Prostate‐related CDUS parameters**		
*Before ejaculation*		
Transitional arteries		
Mean PSV (cm/s)	8.3 ± 1.8	5.0–11.0
Mean acceleration (m/s^2^)	0.58 ± 0.08	0.45–0.71
Mean RI	0.56 ± 0.06	0.48–0.65
Mean PI	0.54 ± 0.12	0.66–1.04
Capsular arteries		
Mean PSV (cm/s)	12.2 ± 1.7	9.0–15.0
Mean acceleration (m/s^2^)	0.69 ± 0.08	0.56–0.82
Mean RI	0.72 ± 0.06	0.64–0.80
Mean PI	0.63 ± 0.12	0.75–1.12
Hyperaemia (%)	0.5	
Periprostatic venous plexus size (mm)	2.9 ± 0.9	1.5–4.5
Periprostatic venous plexus flux velocity (cm/s)	3.8 ± 1.4	2.0–7.0
*After ejaculation*		
Transitional arteries		
Mean PSV (cm/s)	9.8 ± 1.9	6.5–13.0
Mean acceleration (m/s^2^)	0.72 ± 0.09	0.57–0.88
Mean RI	0.66 ± 0.06	0.58–0.75
Mean PI	0.94 ± 0.12	0.76–1.14
Capsular arteries		
Mean PSV (cm/s)	13.9 ± 1.9	10.5–17.0
Mean acceleration (m/s^2^)	0.84 ± 0.09	0.69–1.00
Mean RI	0.84 ± 0.06	0.76–0.92
Mean PI	1.02 ± 0.12	0.84–1.20
Hyperaemia (%)	0.5	
Periprostatic venous plexus size (mm)	3.0 ± 0.9	1.7–4.6
Periprostatic venous plexus flux velocity (cm/s)	5.0 ± 1.4	3.0–8.0

Prostate asymmetry was not observed. Prostate inhomogeneity (of a mild degree) and calcifications were observed in about one out of three subjects, while prostate echogenicity abnormalities were rare. Prostate midline prostatic cysts were uncommon (5%) and small (volume < 0.117 ml and *td* < 5 mm). Ejaculatory duct abnormalities were not observed. The upper limit of prostatic arterial PSV sampled in the transitional zone was 11 cm/s, while that of PVP was 4.5 mm. Interestingly, prostate and SV blood flow‐related parameters, as well as PVP size, showed higher values when measured after than before ejaculation (all *p* < 0.001). Prostate nodules, assessed more carefully in the peripheral zone, were not observed.

Table 4 shows the reference ranges of the SV quantitative parameters (including diameters, volumes and vascular parameters) evaluated before and after ejaculation, and SV echotexture features. In addition, parameters related to SV changes with ejaculation, such as “delta SV total volume” (DSTV) and “SV ejection fraction” (SVEF) (see Methods section) have been reported. Of note, the lower and upper limit of the mean SV‐*apd* after ejaculation, often used to define SV hypotrophy and dilation,^7^ were 6.0 and 16.0 mm, respectively. In addition, the median DSTV was 3.1 ml (while the median semen volume was 3.3 ml), the lower limit of delta SV *ld* and *apd* was 2.0 mm, and the lower limit of SVEF was 20.0%. SV asymmetry, calculated in this study as a difference between SV left and right *ld*, *apd* or volume, considerd after ejaculation, > 10 mm, 5 mm and 3.6 ml respectively, was uncommon (5%). SV echogenicity after ejaculation was normal in more than 90% of men. SV inhomogeneity and roundish anechoic areas were observed before ejaculation in one out of three and one out of six men, respectively. However, their prevalence was significantly lower when assessed after ejaculation (all *p* < 0.001), more specifically, they were halved. SV wall/thickened septa were rare and their frequency did not change with ejaculation, while SV giant cysts were not observed.

**TABLE 4:** Reference range and mean or median values and percentages of the seminal vesicles (SV) colour‐Doppler ultrasound (CDUS) parameters in healthy, fertile men. Data are expressed as mean   ±   SD when normally distributed, as medians (quartiles) for parameters with non‐normal distribution, and as percentages when categorical. The reference range of each testicular parameter has been estimated according to the CLSI Guidelines^48^ as the 5th and the 95th percentiles of its distribution. US, ultrasound; CDUS, colour‐Doppler ultrasound; SV, seminal vesicles; *ld*, longitudinal diameter; *apd*, anterior‐posterior‐diameter; PSV, peak systolic velocity; RI, resistive index; PI, pulsatility index

Seminal vesicles (SV) US parameters	Mean or median values and percentages	Reference range
SV diameters and volume		
*Before ejaculation*		
Right SV *ld* (mm)	48.2 ± 5.8	40.0–57.0
Right SV *apd* (mm)	12.6 ± 3.7	7.5–18.0
Right SV volume (ml)	3.6 (2.1–5.8)	1.3–9.6
Left SV *ld* (mm)	48.0 ± 6.0	40.0–57.5
Left SV *apd* (mm)	12.4 ± 3.7	8.0–18.0
Left SV volume (ml)	3.3 (2.1–5.7)	1.3–9.3
Mean SV *ld* (mm)	48.1 ± 5.4	40.0–56.0
Mean SV *apd* (mm)	12.5 ± 3.5	8.0–18.0
Median SV volume (ml)	3.4 (2.1–5.8)	1.4–9.0
Total SV volume (ml)	6.7 (4.3–11.6)	3.0–18.0
*After ejaculation*		
Right SV *ld* (mm)	45.1 ± 5.8	37.0–54.0
Right SV *apd* (mm)	9.8 ± 3.5	5.7–16.5
Right SV volume (ml)	2.3 (1.4–3.9)	0.5–7.9
Left SV *ld* (mm)	44.6 ± 5.9	36.0–54.0
Left SV *apd* (mm)	12.5 ± 3.7	6.0–17.0
Left SV volume (ml)	2.0 (1.2–3.8)	0.6–8.0
Mean SV *ld* (mm)	44.9 ± 5.4	37.0–53.0
Mean SV *apd* (mm)	9.8 ± 3.3	6.0–16.0
Median SV volume (ml)	1.9 (1.1–3.5)	0.6–6.0
Total SV volume (ml)	3.8 (2.3–7.0)	1.2–12.0
Difference between left and right SV *ld*	2.5 (1.0–5.0)	0.0–10.0
Difference between left and right SV *apd*	1.0 (0.4 –2.3)	0.0–5.0
Difference between left and right SV volume	0.55 (0.14 –1.30)	0.0–3.60
SV asymmetry (%)	5.0	
Delta SV *ld* (mm)	3.3 ± 1.4	2.0–6.3
Delta SV *apd* (mm)	2.7 ± 1.0	2.0–4.8
Delta SV total volume (DSTV) (ml)	3.1 (2.0–4.4)	1.3–6.4
Right SV (ml)	1.6 (0.9–2.4)	0.6–4.2
Left SV (ml)	1.4 (0.9–2.2)	0.6–4.9
Delta SV median volume (ml)	1.6 (1.0–2.3)	0.6–4.7
SV total ejection fraction (SVEF) (%)	43.2 (35.0–52.0)	20.0–58.0
Right SV (ml)	43.2 (18.0–52.0)	18.0–59.0
Left SV (ml)	44.7 (20.0–53.0)	20.0–60.0
Median SVEF (ml)	43.4 (34.0–52.0)	20.0–58.0
Echogenicity before ejaculation (%)		
normoechoic	80.5	
mainly hypoechoic	3.1	
mainly hyperechoic	0.5	
mixed	16.4	
Echogenicity after ejaculation (%)		
normoechoic	90.7	
mainly hypoechoic	1.0	
mainly hyperechoic	0.0	
mixed	8.3	
Inhomogeneity (%)		
before ejaculation	34.2	
after ejaculation	16.7	
Roundish anechoic areas/areas of endocapsulation		
before ejaculation	16.4	
after ejaculation	8.3	
Wall/thickened septa (%)		
before ejaculation	3.6	
after ejaculation	3.6	
Calcifications (%)	0.0	
Giant cysts (%)	0.0	
**SV CDUS parameters**		
SV arteries before ejaculation		
Mean PSV (cm/s)	6.4 ± 1.3	4.0–9.0
Mean acceleration (m/s^2^)	0.60 ± 0.13	0.37–0.88
Mean RI	0.72 ± 0.19	0.40–1.06
Mean PI	1.05 ± 0.29	0.45–1.52
SV arteries after ejaculation		
Mean PSV (cm/s)	6.6 ± 1.3	4.4–9.5
Mean acceleration (m/s^2^)	0.62 ± 0.12	0.39–0.90
Mean RI	0.74 ± 0.20	0.43–1.08
Mean PI	1.07 ± 0.29	0.47–1.55
**Deferential ampullas**		
Right side size (mm)	4.3 ± 0.8	2.5–6.0
Left side size (mm)	4.3 ± 0.9	2.6–6.0
mean size (mm)	4.4 ± 0.6	3.5–6.0

Finally, Table 4 shows the reference range of the deferential ampullas, with an upper limit of 6 mm.

### 3.3 Correlations between TRUS and clinical, seminal and biochemical parameters

Associations between TRUS parameters and clinical, seminal and biochemical characteristics are reported below. Results have been adjusted for confounders (including age, waistline, # EAA Centers, smoking habit, alcohol consumption, physical activity and cFT, when appropriate), unless otherwise specified. In addition, Table 5 shows the correlations between several prostate‐ and SV‐related parameters and abnormalities. Of note, no association between TRUS parameters and time to pregnancy, number of children or history of miscarriage was observed (not shown).

**TABLE 5:** Significant correlations between main prostate‐ and SV‐related parameters and abnormalities. Statistical analysis has been performed considering the variables of the first line as dependent variables and adjusting for confounders (including age, waistline, smoking habit, alcohol consumption, physical activity, calculated free testosterone, number of EAA Centers, and, in addition, sexual abstinence duration when SV were evaluated). (i) Linear regression, (ii) logistic binary regression, or (iii) analysis of covariance (ANCOVA) for comparisons between two groups were applied for multivariate analyses whenever appropriate. PSV, peak systolic velocity; PVP, periprostatic venous plexus, SV, seminal vesicles. Adj. r = adjusted r; OR, odds ratio

	Prostate volume (PV)	Prostate inhomogeneity	Prostate calcifications	Calcification size	Prostatic artery PSV	PVP size	PVP flux velocity	SV volume before ej.	SV volume after ej.	Delta SVtotal volume (DSTV)	SV ejection fraction (SVEF)
**Prostate volume** **(PV)**		OR = 1.10 (1.03–1.17), *p* < 0.01	OR = 1.09 (1.02–1.16), *p* < 0.01	Adj. *r* = 0.404, *p* < 0.001	Adj. *r* = 0.230, *p* < 0.01			Adj. *r* = 0.191, *p* < 0.05	Adj. *r* = 0.215, *p* < 0.05		
**Prostate inhomogeneity**	*F* = 6.9, *p* < 0.01		OR = 3.47 (1.69–7.11), *p* < 0.001	F = 8.0, *p* < 0.01	*F* = 8.7, *p* < 0.005			*F* = 6.2, *p* < 0.05	*F* = 8.4, p <0.05		
**Prostate calcifications**	*F* = 13.4, *p* < 0.001	OR = 3.47 (1.69–7.11), *p* < 0.001			*F* = 13.9, *p* < 0.001	*F* = 8.6, *p* < 0.005					
**Calcification** **size**	Adj. *r* = 0.379, *p* < 0.001	OR = 1.15 (1.02–1.29), *p* < 0.05			Adj. *r* = 0.364, *p* < 0.005	Adj. *r* = 0.230, *p* < 0.05		Adj. *r* = 0.301, p = 0.01	Adj. *r* = 0.276, *p* < 0.05		
**Prostatic** **artery PSV**	Adj. *r* = 0.227, *p* < 0.01	OR = 1.47 (1.14–1.91), *p* < 0.005	OR = 1.38 (1.08–1.78), p = 0.01	Adj. *r* = 0.408, *p* < 0.005							
**PVP** **size**			OR = 1.87 (1.23–2.85), *p* < 0.005	Adj. *r* = 0.236, *p* < 0.05			Adj. *r* = 0.155, *p* < 0.05	Adj. *r* = 0.186, *p* < 0.05	Adj. *r* = 0.193, p< 0.05		
**PVP** **flux velocity**						Adj. *r* = 0.167, *p* < 0.05		Adj. *r* = 0.182, *p* < 0.05	Adj. *r* = 0.249, p< 0.05		
**SV volume before ej**	Adj. *r* = 0.185, *p* < 0.05	O*r* = 5.31 (1.43–19.75), *p* < 0.05		Adj. *r* = 0.310, p = 0.01		Adj. *r* = 0.210, *p* < 0.05	Adj. *r* = 0.171, *p* < 0.05			Adj. *r* = 0.857, *p* < 0.001	
**SV volume** **after ej**.	Adj. *r* = 0.197, *p* < 0.05	OR = 5.22 (1.69–16.17), *p* < 0.005		Adj. *r* = 0.287, *p* < 0.05		Adj. *r* = 0.216, *p* < 0.05	Adj. *r* = 0.262, *p* < 0.05				Adj. *r* = – 0.623, *p* < 0.001
**Delta SV** **total volume** **(DSTV)**								Adj. *r* = 0.397, *p* < 0.05			
**SV ejection fraction** **(SVEF)**									Adj. *r* = – 0.529, *p* < 0.001		

### 3.4 Prostate volume (PV)

Subjects with an enlarged prostate at digito‐rectal examination (DRE) were older (38.8 ± 4.2 vs. 35.1 ± 5.8 years; *p* < 0.05) and had a larger waistline (100.5 ± 13.2 vs. 92.6 ± 8.7 cm; *p* < 0.005) compared to the rest of the sample. As expected, men with an enlarged prostate at DRE showed a higher US‐PV than the rest of the sample (Figure 2B). Interestingly, using ROC curve analysis, an US‐PV > 30 ml identified subjects considered by clinicians as having an enlarged prostate at DRE with an accuracy of 89.6 ± 3.6% (*p* < 0.001) (Figure 2C).

PV was positively associated with age (Figure 2A). Other anthropometric parameters, such as waistline (Figure 2D) and BMI (*r* = 0.236, p = 0.001) were positively associated with PV. Subjects with ≥ 4 components of the MetS construct (AHA/NHLBI classification^20^) showed a higher PV compared to the rest of the sample (Figure 2E). Furthermore, current smokers had a higher PV than non‐smokers (Figure 2F). Besides age, at multivariate analysis, waistline (or BMI) and current smoking were confirmed as additional, independent determinants of PV (Figure 2D and F, respectively).

As expected, PV was positively associated with PSA levels (adj. *r* = 0.151, *p* < 0.05) but not with other biochemical parameters, including total or calculated free T (not shown).

Considering seminal parameters, PV was associated positively with seminal volume and negatively with seminal pH (Figure 2G and H).

**FIGURE 3:**
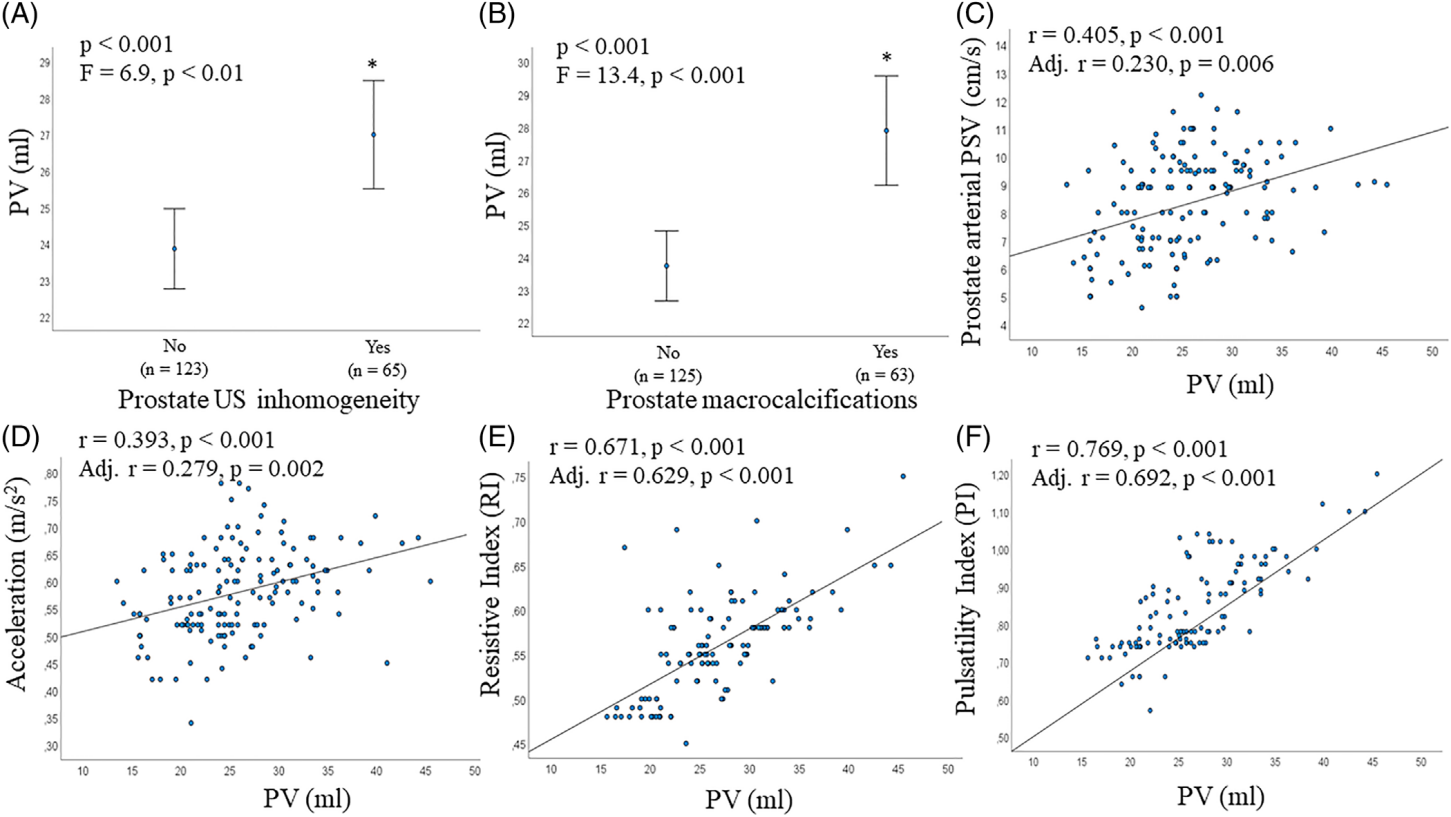
Associations between prostate volume (PV), echo‐texture abnormalities and arterial parameters. A‐B, association between PV, prostate inhomogeneity (A) and macrocalcifications (B). C‐F, association between PV and prostate arterial parameters: peak systolic velocity (PSV) (C), acceleration (D), resistive index (RI) (E), and pulsatility index (PI) (F). (A–F) unadjusted and adjusted (for age, waistline, smoking habit, alcohol consumption, physical activity, cFT levels and # EAA Centers) associations have been reported. *p < 0.001 (unadjusted)

**FIGURE 4:**
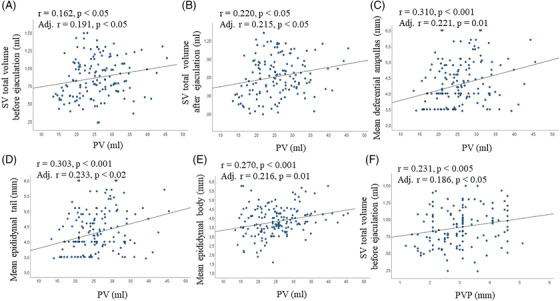
Associations between prostate volume (PV), seminal vesicles (SV) volume, deferential and epididymal size; association between periprostatic venous plexus (PVP) and SV volume. (A and B) Association between PV and SV volume before (A) and after (B) ejaculation. C‐E, associations between PV and mean size of the deferential ampullas (C), tail (D) and body (E) of the epididymes. (F) association between PVP and SV volume before ejaculation. A‐F: unadjusted and adjusted (for age, waistline, smoking habit, alcohol consumption, physical activity, cFT levels and # EAA Centers; in A, B, F also for sexual abstinence duration) associations have been reported

When other TRUS parameters were analyzed, subjects with prostate inhomogeneity or macrocalcifications had a higher PV than the rest of the sample (Figure 3A‐B; Table 5), and PV showed a positive association with the size of the major calcification (Table 5). In addition, PV was positively associated with prostate arterial parameters (including PSV, acceleration, RI and PI) (Figure 3C‐F). Furthermore, PV showed a positive correlation with SV total volume before and after ejaculation (Figure 4A‐B) and, in particular, with SV‐APDs (adj. *r* = 0.216 and adj. *r* = 0.207, both *p* < 0.01). Finally, PV was positively associated with the mean size of the deferential ampullas, and the tail and body of the epididymes (Figure 4C‐E).

### 3.5 Prostate US abnormalities

Men with calcifications were older (37.0 ± 5.6 vs. 35.0 ± 6.2 years; *p* < 0.05), and those with prostate inhomogeneity showed a larger waistline (95.0 ± 13.8 vs. 91.0 ± 8.6 cm; *p* < 0.05) than the rest of the sample. Subjects with calcifications or prostate inhomogeneity more often reported a history of prostatitis than the rest of the sample (OR = 2.40 [1.04‐5.53] and OR = 1.06 [1.01‐1.11]; both *p* < 0.05). In addition, the major calcification in men with leukocytospermia was of a larger size than the rest of the sample (*F* = 11.4, *p* < 0.001). No other associations between prostate US abnormalities and clinical, seminal or biochemical parameters were observed (not shown). In particular, comparing men with and without midline prostatic cysts, we found no difference in seminal parameters (not shown).

Evaluating the correlations between several TRUS parameters, men with prostate inhomogeneity showed prostate calcifications more often as well as a larger size of the major calcification (Table 5). In addition, besides the aforementioned associations between prostate calcifications or inhomogeneity and PV (see above and Table 5), men with prostate calcifications or inhomogeneity showed a higher prostatic arterial PSV, and those with calcifications also had a larger PVP size, than the rest of the sample (Table 5). Of note, the size of the major calcification showed a positive correlation with prostatic arterial PSV (Table 5). Furthermore, calcification size and prostate inhomogeneity showed a positive correlation with SV total volume before and after ejaculation (Table 5).

### 3.6 Prostate‐related vascular parameters

Prostatic arterial parameters (PSV, acceleration, RI and PI), both in the transitional and capsular zone, had positive associations with each other (all *p* < 0.001). In addition, each arterial parameter evaluated in the transitional zone showed a positive correlation with the same parameter measured in the capsular zone (all *p* < 0.001). Hence, we refer below to parameters assessed in the transitional zone. Prostatic arterial PSV showed a positive association with age and waistline (*r* = 0.211, *p* < 0.01 and *r* = 0.253, *p* < 0.005, respectively). In addition, men with leukocytospermia showed a higher prostatic arterial PSV than the rest of the sample (*F* = 4.6, *p* < 0.05). According to the results reported above, prostatic arterial PSV was associated with an increased risk of enlarged PV, prostate inhomogeneity, macrocalcification detection and larger size (Table 5). Similar associations were found considering acceleration, RI and PI (not shown).

PVP size and flux velocity were positively associated with each other (Table 5). Men with abnormal seminal viscosity showed a higher PVP flux velocity than the rest of the sample (*F* = 15.0, *p* < 0.001). PVP size correlated positively with prostate calcifications (Table 5) and with SV total volume before (Figure 4F) and after ejaculation (Table 5). Similar figures were observed for PVP flow velocity (Table 5). In addition, PVP size showed a positive association with SV arterial PSV (adj. r = 0.397, *p* < 0.05).

No other association between prostate‐related vascular parameters and clinical, seminal or biochemical features were observed (not shown).

### 3.7 Seminal vesicles (SV) volume

**FIGURE 5:**
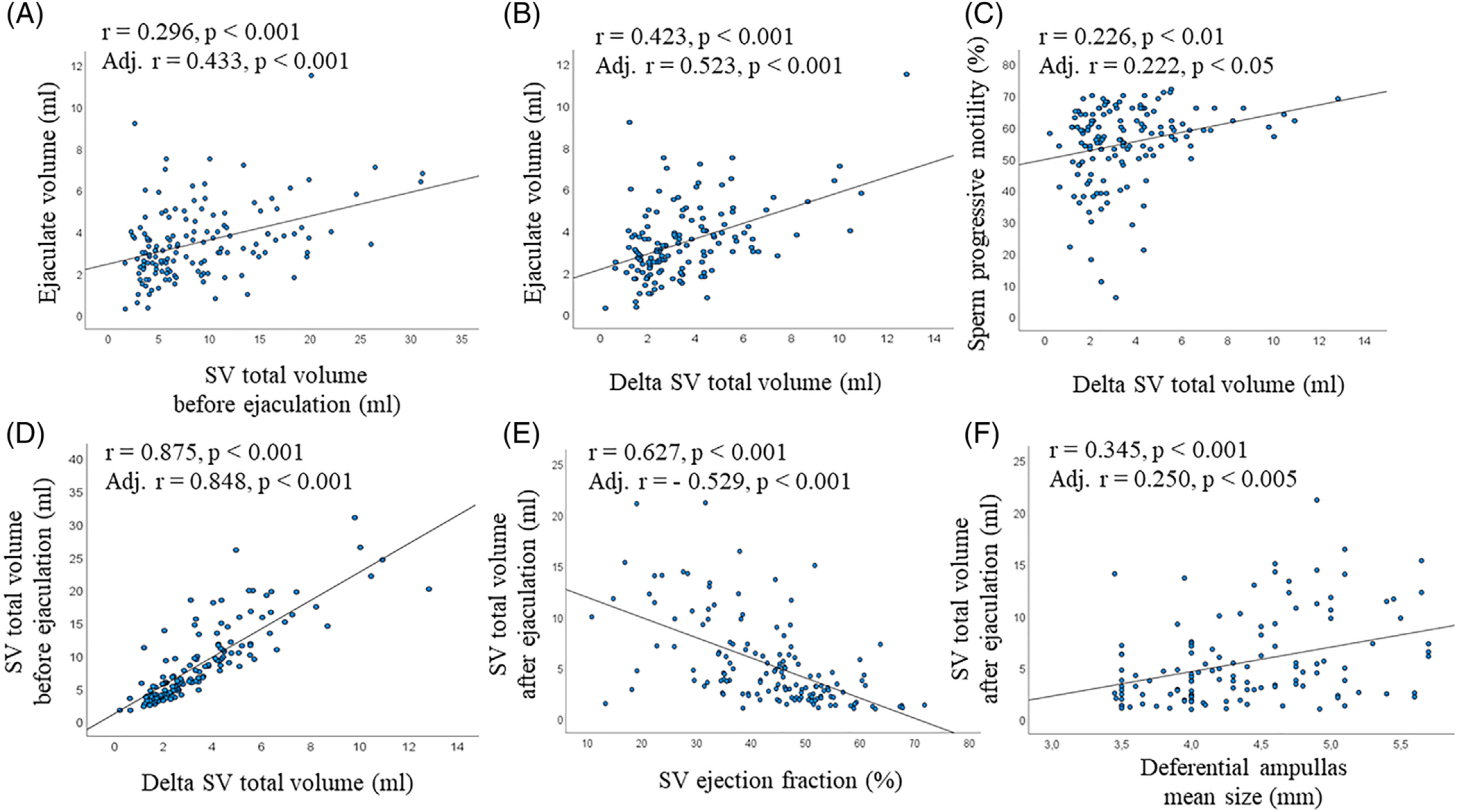
Relevant associations of parameters related to SV volume before and after ejaculation. (A) Association between SV total volume and ejaculate volume. (B–D) associations between delta SV total volume and ejaculate volume (B), sperm progressive motility (C) and SV total volume before ejaculation (D). (E–F) Association of SV total volume after ejaculation, SV ejection fraction (E) and mean size of the deferential ampullas (F). (A–F) unadjusted and adjusted (for age, waistline, smoking habit, alcohol consumption, physical activity, cFT levels, # EAA Centers and sexual abstinence duration) associations have been reported

SV total volume before and after ejaculation was positively associated with sexual abstinence duration (*r* = 0.197 and *r* = 0.174, respectively; both *p* < 0.05). No association was found betweeen SV total volume and age, waistline, lifestyle parameters, hormonal (including total and calculated free T levels) and glyco‐metabolic parameters (not shown). In the adjusted model, including sexual abstinence duration as a further covariate, we observed a positive association between SV total volume before ejaculation and ejaculate volume (Figure 5A). In addition, men with leukocytospermia or bacteriospermia had a higher SV total volume before ejaculation than the rest of the sample (*F* = 4.1 and *F* = 5.8, respectively; both *p* < 0.05).

As reported above, SV total volume was positively associated with PV, prostate inhomogeneity, major calcification size, PVP size and flux velocity (see Table 5). In addition, men with SV septa, inhomogeneity or roundish anhecoic areas had higher SV volumes before and after ejaculation than the rest of the sample (all *p* < 0.05).

### 3.8 Delta SV total volume (DSTV) and SV ejection fraction (SVEF)

DSTV was positively associated with sexual abstinence duration (*r* = 0.224, *p* < 0.01). In the adjusted model, including sexual abstinence duration as a further covariate, DSTV was positively associated with ejaculate volume and sperm progressive motility (Figure 5B‐C). Finally, DSTV was positively associated with SV volume before ejaculation (Figure 5D)

SVEF was negatively associated with age and waistline (*r* = ‐0.165 and *r* = ‐0.206, respectively; *p* < 0.05) and with SV volume after ejaculation (Figure 5E).

No correlations between DSTV or SVEF and other clinical, biochemical, seminal and CDUS parameters were observed (not shown).

### 3.9 SV‐CDUS abnormalities

Subjects with SV roundish anhecoic areas before ejaculation showed a higher ejaculate volume (*F* = 6.9, p = 0.01), DSTV and SV volume before ejaculation (*F* = 7.5, *p* < 0.01 and *F* = 6.0, *p* = < 0.05, respectively) than the rest of the sample. Considering SV vascular parameters, only arterial PSV showed a positive association with PVP size (see above). No correlation between other SV‐CDUS and clinical, seminal, biochemical or MGT‐CDUS parameters were observed.

### 3.10 Deferential ampullas

The mean deferential ampullas diameter was positively associated with PV (see above), prostatic arterial PSV (adj. *r* = 0.300, *p* < 0.001), SV volume before (adj. *r* = 0.300, *p* < 0.001) and after (Figure 5F) ejaculation, and, at scrotal level, with the mean size of proximal vas deferens, epididymal body and tail (adj. *r* = 0.282, *p* < 0.05; adj. r = 0.319, *p* < 0.001; adj. *r* = 0.208, p <0.05). No correlation between deferential ampullas and other MGT‐CDUS, clinical, seminal or biochemical parameters were observed (not shown).

## 4 DISCUSSION

In this study we have assessed the reference ranges and CDUS characteristics of the prostate and SV of 188 subjects derived from a multinational cohort of 248 healthy, fertile men,^20^ who accepted to undergo TRUS before and after ejaculation. In addition, we reported and herein discuss the correlations of the TRUS parameters with clinical, seminal and biochemical characteristics evaluated on the same day.

Investigator meetings organized by the EAA US consortium before enrolling healthy, fertile men^20^ led to the definition of the Standard Operating Procedures (SOPs) for the assessment of TRUS qualitative and quantitative parameters. They have been discussed extensively on the EAA website (http://www.andrologyacademy.net/studies19) and here are reported in the Methods section. This careful methodological alignment and the agreement reached by the sonographists of the different EAA Centers are reflected in the high inter‐ and intra‐operator comparability. In fact, we found a relatively low coefficient of variation (< 10)^47^ and a high concordance rate for quantitative and qualitative TRUS parameters according to the National Association of Testing Authorities (NATA) criteria.^46^ In our opinion, following the CDUS SOPs proposed by the EAA US consortium in clinical practice would help to reduce the current operator‐dependent differences among sonographers.

In healthy, fertile men we found a mean PV of 25.0 ± 6.3 ml, with a lower and upper limit of 15 and 35 ml, respectively. The same reference range was observed in selected eugonadal men without central obesity, strengthening the concept of “normative limits” in a healthy population. In addition, the PV reference range in three age decades between 20 and 50 years has been reported. Previous studies suggested a PV > 30 ml^49^ to indicate an initial prostate enlargement, and > 60 ml^50^ to indicate a severe enlargement in aging men with benign prostatic hyperplasia (BPH). The EAA US study reports evidence‐based US‐PV normative limits defining, in men of reproductive age, an enlarged (> 35 ml) or small (< 15 ml) prostate at TRUS. In addition, we derived a simple mathematical formula (1/3 age + 15) to calculate, in healthy young/adult men, the age‐adjusted normative mean PV. The aforementioned formula and thresholds can be useful in clinical practice to derive the expected average PV by age and identify an initial prostatic hyperplasia or hypotrophy. In particular, the detection of a small prostate at TRUS can corroborate a DRE suspicion of prostate hypotrophy, eventually suggesting T deficiency.^7^ On the other hand, this study shows that TRUS often confirms the DRE suspicion of prostatic enlargement. Enlarged prostate at DRE as well as US‐PV were associated positively with age and waistline, and US‐PV also with severe MetS. These results are in line with previous studies,^24^, ^29^, ^51^, ^52^, ^53^, ^54^, ^55^ reporting that age^51^, ^52^, ^53^ and waistline/MetS^24^, ^29^, ^53^, ^54^, ^55^, ^56^ are independent risk factors for prostate enlargement. Interestingly, those previous studies performed on young/adult men evaluated subjects with couple infertility and overweight/obesity^29^, ^55^, ^56^ or MetS,^24^, ^29^, ^56^ while the EAA US study investigated fertile men with a low metabolic burden,^20^ confirming the aforementioned associations in a healthy, fertile population. In addition, in this study, current smoking was associated with prostate enlargement. Previous studies reported conflicting results on this topic, supported by different pathophysiological models.^53^, ^57^ A recent systematic review and meta‐analysis^57^ underlined a trend of BPH risk in current smokers compared to nonsmokers, although no significant association between smoking and BPH was found. Hence, further studies are needed to elucidate this point.

Evaluating biochemical parameters, PV was positively associated with PSA levels but not with other parameters, including total or calculated free T. The association between PV and PSA levels is well established.^6^ Conversely, although the prostate is an androgen‐dependent gland,^7^, ^16^, ^17^, ^18^, ^44^, ^51^ the association between T levels and PV is debated.^52^, ^58^ In fact, previous studies reported a positive effect of T on PV in hypogonadic subjects under androgen replacement therapy,^7^, ^16^, ^17^, ^18^, ^51^ but not during continued (> 24 months) T treatment^52^ or in aging men with BPH.^58^ This phenomenon could be explained with the androgen receptor “saturation” hypothesis,^59^ postulating that the human prostate is sensitive to androgens when the receptor is not saturated, as in severe hypogonadism, but rather insensitive in normal (eugonadism) or even subnormal (mild hypogonadism) conditions.^60^ Accordingly, the healthy, fertile cohort was made‐up almost entirely of eugonadic subjects.^20^ In addition, the relatively narrow range of T levels and PV values in our cohort^20^ could negatively affect the ability to detect an association between these two parameters.

Considering seminal parameters, PV was associated positively with seminal volume and negatively with seminal pH. These results are in agreement with the well‐known contribution of the prostate to semen volume and pH by secreting prostatic fluid, an acidic secretion which makes up ∼30% of the total ejaculate.^5^, ^7^, ^61^

Evaluating US correlates of PV, a large PV was often associated with prostate inhomogeneity and macrocalcifications, even after adjusting for confounders including age and waistline. Hence, an increased PV could reflect inflammatory or metabolic insults leading to inhomogeneity or calcifications regardless of age and waistline, which are notoriously associated with these findings.^24^, ^29^ However, considering the cross‐sectional nature of this study, we cannot exclude that inhomogeneity per se, reflecting an inflammatory/edematous state of the prostate,^7^, ^28^, ^62^, ^63^, ^64^ or the presence of macrocalcifications, can lead to PV enlargement.

In line with both of the aforementioned scenarios, PV was positively associated with prostate arterial parameters. Previous studies reported that prostatic hyperemia^7^, ^11^ and increased prostatic arterial PSV in both aging men with BPH^33^ and younger subjects with couple infertility^12^ represent signs of prostate inflammation. In this study, the association between PV and prostatic arterial PSV in the normal range could suggest an incipient/initial prostatic inflammatory state in men with a larger PV. Accordingly, men with overweight/obesity^29^, ^55^ or MetS,^24^ characterized by a systemic, low‐grade inflammatory state, showed increased PV and prostatic arterial PSV compared to men with normal weight or without MetS, respectively. Of note, in healthy, fertile men, the upper limit of prostatic arterial PSV was 11 cm/s. In a previous study evaluating men with couple infertility,^12^ a PSV > 11 cm/s identified subjects with moderate‐severe prostatitis‐like symptoms, indicating current prostate inflammation. These data are corroborated by another study^13^ reporting an association between prostatitis‐like symptoms, increased prostatic arterial PSV and acquired premature ejaculation, of which prostate inflammation is considered an organic cause. Overall, these data suggest that in young/adult men (< 50 years) a prostatic arterial PSV < 11 cm/s can be considered “normal”, while higher values indicate prostate inflammation. Of note, to standardize the use of prostatic arterial PSV as a parameter for identifying inflammation, it must be measured before ejaculation. In fact, we found that PSV increases significantly after ejaculation, according to a previous report.^65^ Interestingly, we found also significant associations between PV and acceleration, RI and PI, the latter two being particularly strong, regardless of several confounders. As expected, in this study, the RI assessed in the prostatic arteries before ejaculation in young/adult men was lower than that reported in BPH^33^, ^35^ or normal prostates^35^ of elderly men, while no previous study has reported reference ranges of prostate arterial acceleration and PI. Acceleration is a parameter strongly related to PSV and systolic rise time,^34^ which increases at the level of an arterial stenosis, as documented in carotid,^66^, ^67^ renal and lower extremity arteries.^67^ RI is very sensitive in evaluating peripheral vascular resistance and is one of the most reliable indicators of vascular damage in the prostate.^33^ It has been reported that BPH patients show a higher RI than men with normal prostates, suggesting that prostate vascular damage leads to tissue hypoxia inducing fibromuscular overgrowth and BPH.^33^ PI is a parameter reflecting resistance to blood flow, associated with microvascular lesions, as documented in the brain, kidneys, uteroplacental circulation and clitoris.^36^ Overall, these data suggest that in healthy, fertile men, the association between the aforementioned arterial parameters and PV may reflect an initial prostate microvascolar damage, possibly secondary to inflammatory or metabolic insults, that could act as a trigger for a subclinical, early‐onset form of BPH. On the other hand, it could be hypothesized that an enlarged prostate could exert compression on prostate arteries, leading to an increase in vascular resistance and CDUS arterial parameters. Accordingly, in a previous study,^35^ the authors suggested that the prostate, enclosed by its capsule, represents a closed system and that hypertrophy, leading to an increase of intraprostatic pressure, may lead to vascular compression. Whatever the correct hypothesis is, our data indicate that clinicians should pay attetion to parameters strongly related to PV, such as RI and PI, and carefully evaluate them in future studies.

Finally, PV was positively associated with SV total volume before and after ejaculation, as well with the mean size of deferential ampullas and epididymes. These results suggest that PV enlargement could exert compression on ejaculatory ducts leading to a slight dilation of upstream organs, similarly, albeit in a milder way, to what has been observed in men with partial or complete obstruction of the ejaculatory ducts.^5^, ^7^, ^43^, ^68^, ^69^

Evaluating prostate US abnormalities, one out of three subjects had calcifications and inhomogeneity. Previous studies attributed these anomalies to inflammatory outcomes or chronic prostate inflammation.^7^, ^23^, ^27^, ^28^, ^62^, ^63^, ^64^ Furthermore, some authors included these US findings in a broader context, “male accessory gland infections” (MAGI).^27^, ^28^, ^70^ Subsequently, some authors reported that prostate inflammation or MAGI could be associated with poor seminal parameters^70^ and male infertility,^71^ suggesting that TRUS detection of calcifications and inhomogeneity could be useful in the male infertility workup. However, a possible negative impact of prostatic inflammation on seminal quality and male fertility is still under debate.^12^, ^70^, ^72^ Therefore, the relationship between prostatic inflammation, TRUS aforementioned abnormalities, sperm parameters and infertility remains controversial. Accordingly, the detection of the aforementioned prostate US anomalies plays a small role in the clinical management of male infertility.^7^ These concepts are supported by the high prevalence of prostate calcifications and inhomogeneity in healthy, fertile men, which suggests that these findings have a marginal impact on fertility. They could rather represent outcomes of previous infections, often subclinical, possibly contracted during sexual activity over time, or inflammation related to the subject's metabolic state. In fact, in this study, the prevalence of prostate calcifications and inhomogeneity increased with age and waistline, respectively. Accordingly, some authors,^73^, ^74^ evaluating healthy men, found that about half had prostatic calcifications, with frequency increasing with age.^73^ However, as a corollary, several studies report that prostatic calcifications are associated with, and maintain, a chronic inflammatory state in the prostate.^7^ Accordingly, the present study reports an association between calcifications and leukocytospermia or increased prostatic arterial PSV, seminal and CDUS signs of inflammation,^7^, ^23^, ^27^ respectively.

Among prostate US abnormalities, midline prostatic cysts were rare (5%) and small (volume < 0.117 ml and *td* < 5 mm), showing no impact on seminal parameters. A previous study^30^ reported that in men with a severe infertility factor, midline prostatic cysts were more frequent (up to 15%) and larger than those observed in fertile men. In particular, a cyst volume > 0.117 ml identified men with severe oligo‐ or azoo‐spermia with ∼75% accuracy, and almost half of these patients had a volume > 0.250 ml with a *td* > 1 cm.^30^ Hence, midline prostatic cysts, frequent and large in infertile men, are detectable also in fertile men, but in the latter they are rarely observed and small, exerting no negative impact on fertility. Conversely, ejaculatory duct abnormalities were not detected in fertile men, supporting their negative role on male fertility according to previous studies.^68^, ^69^, ^75^

For the first time, we report the reference range of the periprostatic venous plexus (PVP), identifying an upper limit of 4.5 mm. Some authors previously suggested to define “PVP dilation” as “a surface of the largest venous section > 150 mm^2^” ^76^ (methodologically scarcely replicable), or a PVP diameter > 3 mm^77^ (study in Japanese language) or > 4 mm^64^ (value proposed but not evidence‐based). The EAA US study indicates as normal, in an evidence‐based way, a PVP‐*apd* < 4.5 mm. It is noteworthy that the size and blood flow of the periprostatic veins were higher ​​when measured after than before ejaculation. Hence, similarly to what has been reported for the prostatic arteries,^65^ the standardization of the measure of PVP‐related parameters requires their assessment before ejaculation. In this study, PVP size and its flow velocity were positively associated. PVP size was positively associated with seminal abnormal viscosity and the presence and size of prostatic calcifications, suggesting a link between a larger PVP and prostatic chronic inflammation. This result is in line with a previous study^23^ reporting a larger PVP in subjects with MAGI than in those without. In addition, some authors^14^ reported an enlarged PVP as a sign associated with prostate inflammation, together with prostatic calcifications, hypoechoic echotexture and elevated seminal interleukin‐8, a proinflammatory cytokine suggestive of MGT^78^ and, in particular, prostate^23^, ^79^ inflammation. Of note, in that study^14^, an enlarged PVP has been suggested as indicative of intrapelvic congestion underlying prostatic inflammation. In the present study, PVP size and flow velocity were also positively associated with SV volume and arterial PSV. It can be speculated that the communication between the prostatic and the vesicular venous systems through the vesicular veins^80^ might justify a venous flow from the PVP to the SV, leading to a SV inflammatory state, documented by their enhanced arterial PSV and underlying their volumetric increase. However, this hypothesis needs to be confirmed.

In this study we report, for the first time, evidence‐based upper and lower limits of the SV diameters and volume, before and after ejaculation, and criteria to define SV asymmetry. In particular, the upper and lower limits of the mean SV‐*apd* after ejaculation, often used in literature to define the thresholds for SV dilation^27^, ^68^, ^69^, ^75^, ^81^ or hypotrophy,^27^, ^82^, ^83^ respectively, were 16 and 6 mm. Previous studies have proposed an *apd* > 14 mm^27^ or > 15 mm^68^, ^69^, ^75^, ^81^ to indicate SV dilation, suggestive of partial or complete ejaculatory duct obstruction. The EAA US study proposes a SV‐*apd* threshold after ejaculation of 16 mm, possibly identifying ejaculatory duct sub‐obstruction with greater accuracy. On the other hand, some authors proposed an *apd* < 7 mm^27^ or < 5 mm,^82^ or a *ld* < 25 mm,^83^ to indicate SV hypotrophy. The EAA US consortium proposes a SV‐*apd* threshold of 6 mm, which is halfway between those previously suggested,^27^, ^82^ and a *ld* threshold of 36 mm, which differs from what has been previously suggested, which, however, was based on the author's personal opinion.^83^ In addition, this study reports, for the first time, SV volume reference range. According to SV volume lower and upper limits, considered after ejaculation to avoid the bias of sexual abstinence, the volume of a single SV < 0.6 ml or > 6 ml could be used to define SV hypotrophy or dilation, respectively. However, the proposed volumetric thresholds need to be confirmed by future studies.

Examining the SV before and after ejaculation, the lower limit of “delta” SV *ld* and *apd* was 2 mm and the median “delta SV total volume” (DSTV) was 3.3 ml, slightly lower than the median seminal volume (3.1 ml) of the cohort studied. These data suggest that, in healthy, fertile men, the normal SV emptying with ejaculation can be defined by a reduction in the SV diameters of at least 2 mm, and that the contribution of SV to the ejaculate volume can reach up to 90%, amplifying the role of SV in determining the amount of the ejaculated seminal fluid.^5^, ^7^, ^61^ Accordingly, the DSTV was positively associated with the ejaculate volume. In addition, DSTV correlated positively with progressive sperm motility. The latter result is in line with the observations of some authors,^84^, ^85^ who reported that SV secrete substances such as potassium, bicarbonate, prostaglandins and prolactin, capable of improving sperm motility in vitro, and that reduced SV function is associated with asthenospermia. Furthermore, DSTV correlated positively with SV total volume before ejaculation, and the latter with ejaculate volume, suggesting that the extent of SV contraction and their contribution to ejaculate volume is proportional to the initial SV size. This hypothesis makes DSTV resemble the “systolic volume” (or “systolic stroke” or “ejection volume”) of the left ventricle, which follows the Frank‐Starling law, whereby the energy of the contraction of the left ventricle is proportional to the initial length of the myocardial fibers (preload).^86^ On the other hand, we also evaluated another parameter, the “SV ejection fraction” (SVEF). It has been previously reported^5^, ^7^ that SVEF represents a useful indicator of ejaculatory duct sub‐obstruction, identifying, for a value ​​< 21.6%, males of infertile couples with reduced seminal volume (<1.5 ml) and pH (<7.2). The lower SVEF limit observed in fertile subjects was 20%, similar to that reported above in infertile men with distal sub‐obstruction.^5^, ^7^ Of note, SVEF was negatively associated with SV total volume after ejaculation. It could be hypothesized that this association depends on a resistance to SV emptying (SV or ejaculatory duct sub‐obstruction) which does not allow the SV to empty themselves completely. This hypothesis suggests that SVEF resembles the left ventricular “ejection fraction”, which indicates the effectiveness of the heart's pump function/inotropy, reduced in the presence of a significant afterload.^86^ Accordingly, the SVEF could reflect the effectiveness of the SV contractile function, reduced in the presence of resistance downstream, at the level of the ejaculatory ducts. In line with this hypothesis, SV volume after ejaculation was positively associated with the mean size of the deferential ampullas, suggesting that a sub‐obstruction of the ejaculatory ducts can involve the entire seminal path, with an upstream dilation of the SV and the deferential ampullas.^5^, ^7^ Of note, in this study, deferential ampullas showed an upper limit of 6 mm, in agreement with previous reports.^7^ All aforementioned hypotheses, formulated in a setting of healthy, fertile men with no clear signs of ejaculatory duct obstruction, although supported by previous observations in males of infertile couples,^5^ must be confirmed in further studies including men with proven ejaculatory duct obstruction.

Among the SV‐US abnormalities, the most interesting were the “roundish anechoic areas”. The prevalence of these areas, observed before ejaculation in one out of six men, was halved after ejaculation. A similar reduction with ejaculation was previously observed in infertile men.^5^ In addition, in this study, the detection of anechoic areas before ejaculation was positively associated with the ejaculate volume. Overall, these observations suggest that the “roundish anechoic areas” represent liquid areas expelled from the SV with ejaculation, and that when present in the SV after ejaculation may indicate incomplete SV emptying. Previous studies have reported a frequency of anechoic areas in infertile men nearly double^5^ that of the fertile men of this cohort, and suggested that such areas can indicate SV stasis,^7^, ^43^ chronic inflammation and MAGI.^7^, ^27^, ^28^ The EAA US study suggests that these areas must be evaluated after ejaculation, to avoid their overestimation and an excessive diagnosis of MAGI, with relevant aspects both from a clinical and scientific point of view. Finally, we also observed that the frequency of SV thickened septa, another parameter associated with chronic SV inflammation,^7^, ^27^, ^28^ was rare in healthy, fertile men, while we did not observe the presence of SV giant cysts, often observed in subjects with genitourinary anomalies.^7^, ^44^

## 5 CONCLUSIONS

In this study we have assessed the reference ranges and CDUS characteristics of the prostate and SV investigated in 188 young/adult subjects, derived from a multinational cohort of 248 healthy, fertile men,^20^ who underwent TRUS before and after ejaculation. In addition, we reported and discussed the correlations of the TRUS parameters with clinical, seminal and biochemical characteristics evaluated on the same day. To standardize the assessment of the prostate‐vesicular parameters, we suggest the evaluation before ejaculation, except for the study of SV anechoic areas and emptying characteristics as well as signs of obstruction, which should be performed also after ejaculation. The present findings in healthy, fertile men will help in better understanding the pathophysiology of semen abnormalities and male infertility and the significance attributed to specific TRUS findings.

## CONFLICT OF INTEREST

None.

## Supporting information

Supporting Information
