# Supplementary material for: The European Academy of Andrology (EAA) ultrasound study on healthy, fertile men: Prostate‐vesicular transrectal ultrasound reference ranges and associations with clinical, seminal and biochemical characteristics
Source: Andrology. 2022 Jul 19;10(6):1150–71. doi: 10.1111/andr.13217 (PMC9544532; doi:10.1111/andr.13217)
Supplement: Supplementary file 1 — Supporting Information [file ANDR-10-1150-s001.docx]

| **EAA Center** | **Ultrasonographic console** | **Transrectal probe** |
| --- | --- | --- |
| Ancona | HD7 XE (Koninklijke Philips N.V., Amsterdam, Nederlands) | intracavitary probe C8-4v |
| Barcelona | ACUSON S2000™ (Siemens Medical Solutions USA, Inc.) | transrectal transducer EC9-4 |
| Cairo | Mindray DC-3 (Shenzhen, China) | transrectal biplanar transducer 6LB7 |
| Catania | MyLab Class C (Esaote SpA, Genova, Italy) | transrectal biplanar probe (linear transducer TRT33L 3–13 MHz; convex transducer TRT33C 3–13 MHz; Esaote SpA, Florence, Italy) |
| Florence | MyLab Class C (Esaote SpA, Genova, Italy) | transrectal biplanar probe (linear transducer TRT33L 3–13 MHz; convex transducer TRT33C 3–13 MHz; Esaote SpA, Florence, Italy);  ‘end fire’ probe (EC123 3–9 MHz, field of view 50–200°; Esaote SpA, Florence, Italy); |
| Giessen | BK Medical Pro Focus (BK Medical, Quickborn, Germany) | transrectal probe 8808e |
| Halle | SONOLINE G60 S (Siemens Medical Solutions, Erlangen, Germany) | transrectal probe BE9-4 |
| L’Aquila | Logiq7 (General Electric, Healthcare, WI, USA) | biplane endocavity transducer BE9C |
| Muenster | BK Medical - Pro Focus Ultraview 2202 (BK Medical, 2730 Herlev, Denmark) | endocavity biplane probe 8808 |
| Rome | Philips IU22 unit (Philips, Bothell, WA, USA) | C9-5EC endocavitary probe |
| Tartu | Flex Focus 400 (BK Ultrasound, 2730 Herlev, Denmark) | prostate biplane probe 8808e |

**Supplementary Table.** Ultrasonographic equipments used by the different EAA Centers.
